# Utilization of Mental Health Support Systems in the Aftermath of Disasters in Japan: Statistical Data of the Miyagi Disaster Mental Health Care Center

**DOI:** 10.3390/ijerph191710856

**Published:** 2022-08-31

**Authors:** Naru Fukuchi, Shusaku Chiba

**Affiliations:** 1 Department of Psychiatry, Tohoku Medical and Pharmaceutical University, Sendai 981-8558, Miyagi, Japan; 2 Miyagi Disaster Mental Health Care Center, Sendai 980-0014, Miyagi, Japan; 3 Department of Child Psychiatry, Iwate Medical University Hospital, Yahaba 028-3695, Iwate, Japan; 4 Faculty of Education, Graduate School of Education, Tohoku University, Sendai 980-8576, Miyagi, Japan

**Keywords:** mental health, earthquake, tsunami, Japan, Miyagi prefecture, post-disaster mental health service

## Abstract

Large-scale natural disasters have a significant effect on residents’ mental health. The Miyagi Disaster Mental Health Care Center (DMHCC) was established as a long-term mental health care center in response to the 2011 Great East Japan Earthquake and Tsunami (GEJE). Although six DMHCCs have been established in Japan, their exact role and functioning are still unclear. This study aimed to explore which population used the center in each recovery phase. Logistic regression was performed to identify the residents’ characteristics according to the consultation pathways using the data collected by the Miyagi DMHCC. These data included personal information of the residents who were supported by the center from 2013 to 2018. The working-age unemployed men sought help by themselves, and the isolated older females were supported by home visits through the health survey. Long-term mental health care centers should observe community recovery and provide appropriate support. The implications of this result and future research directions are discussed.

## 1. Introduction

Natural disasters such as floods, earthquakes, hurricanes, and fires have significant effects on human beings’ health. Studies report that major disasters have significant negative effects on survivors’ socio-emotional functioning and mental health [1,2,3]. Although a few issues are diagnosed as disorders, others are not. A recent meta-analysis of 27 studies concluded that the prevalence of post-traumatic stress disorder (PTSD) and depression increase significantly after natural disasters [4]. Similarly, North et al. [5] report that among disaster survivors, the prevalence of other anxiety disorders is much lower than that of PTSD and major depression. Somatic symptoms are also common in residents who have experienced disasters; these include sleep disturbances, musculoskeletal pain, and loss of appetite [6,7]. Sleep disturbance following trauma is common and, when left untreated, can occur up to several years after a natural disaster, due to residual anxiety [8]. Musculoskeletal pain (low back pain, shoulder pain, and knee pain) was found to be common after the Great East Japan Earthquake and Tsunami (GEJE) [9]. Additionally, musculoskeletal pain and sleep disturbance frequently occur simultaneously.

### 1.1. Mental Health Needs Depend on Recovery Phases

In the aftermath of a large-scale natural disaster, the psychological needs of a community change with each recovery stage [10]. McFarlane [11] indicates that medical services tend to be prioritized during the rescue phase, whereas mental health services are in high demand in subsequent phases. Although only a few residents need psychological support in the immediate aftermath of a disaster, mental health support teams must make preliminary assessments of their potential long-term needs. Houston et al. [12] reported that social support for residents with probable PTSD and depression was poor, and these residents did not use mental health resources effectively after the catastrophic 2011 Joplin tornado. Similarly, Brown et al. [13] reported that several residents were at risk of suicide, even after receiving mental health care in community settings (residents’ homes, schools, churches, and mobile units) during the aftermath of a series of hurricanes in Florida. These occurrences demonstrate that teams of well-trained professionals who can screen at-risk individuals are essential, and that active outreach based on screening is necessary.

Post-disaster mental health services have been established in many cases and various types of mental health support have been provided after many disasters around the world; these include the 2004 tsunami in the Maldives [14], Hurricane Katrina in the United States in 2005 [10], the 2017 flooding in Peru [15], and the 2010 earthquake in Haiti [16]. These studies all reported that depression and PTSD occurred for numerous years after the disaster; moreover, residents with existing mental illnesses—such as schizophrenia and depression—reported worsening or recurrence of their condition. Furthermore, North [2] reports that a long-term mental health support system is needed to support residents with vulnerabilities and reconstruct a community-based mental health system. Adults can have significant mental health needs that can exist for 6–18 months after a disaster [12]. Therefore, long-term programs aimed at monitoring, assessment, and outreach are necessary for an extended period after a major disaster. Although the importance of long-term mental health support has become widely known, it is unclear which populations need support, which methods are effective for the residents’ mental health in each phase, and how local governments can operate a long-term mental health center, depending on the recovery phases.

### 1.2. The Post-Disaster Mental Health System in Japan

First, the history of the emergency mental support system in Japan should be reviewed. The emergency mental health support system and the long-term mental health support system were established after two major disasters in Japan: the 1995 Hanshin-Awaji earthquake (magnitude 7.3; 6434 deaths) and the 2011 GEJE (magnitude 9.0; 19,630 deaths [17]). Prior to this, various groups formed ad-hoc support teams that provided services in the disaster-affected areas. These efforts were marred by unfortunate incidents, in which rescue teams caused inadvertent damage to the mental health of survivors, owing to the absence of appropriate skills to carry out their operations, or confusion regarding the prevailing circumstances [18]. Based on these experiences, mental health care teams consisting of psychiatrists, nurses, and social workers were formed and dispatched to the GEJE disaster-affected areas in 2011. The need for well-trained professionals who specialize in emergencies was recognized. Consequently, Japan’s Ministry of Health, Labor and Welfare set up a formal system comprising a Disaster Psychiatric Assistance Team in 2013 [19,20,21]. However, most of the previous literature mainly focuses on the acute recovery phase and although health outcomes most frequently assessed were mental disorders, the long-term strategies have been still unclear [22,23].

In Japan, local governments and public health centers play an important role in supporting communities in their long-term recovery, in the wake of disasters. However, these facilities could not sufficiently support communities, as the scale of the disaster was overwhelming. The Japanese government and the affected municipalities, therefore, established new organizations, such as the Disaster Mental Health Care Centers (DMHCC), which specialized in reconstructing local mental health systems. For example, the Hyogo DMHCC was established in 1995 in response to the Hanshin-Awaji earthquake, while the Niigata DMHCC was established in 2004 in response to the Niigata-Chuetsu earthquake [24,25]. Similarly, three DMHCCs were established in the prefectures of Iwate, Miyagi, and Fukushima in the aftermath of the GEJE. Although all the DMHCC’s activities have been reported in Japan, the active data have not been sufficiently analyzed; therefore, the factors related to long-term mental health systems are still unclear.

### 1.3. The GEJE and the Miyagi DMHCC

Miyagi is the largest prefecture in the Tohoku region, with a population of 2,323,224 people in 2011. The Tohoku region is a relatively rural area in Japan and the mental health indicators among its residents are worse than those of other regions. In 2011, the Tohoku region had a suicide rate of 27.1 per 100,000 people [26]. Although the main industries are agriculture and fishing, the region had a severely aging population with an aging rate of 22.2% in 2011 and a shortage of labor population [27].

**Figure 1:**
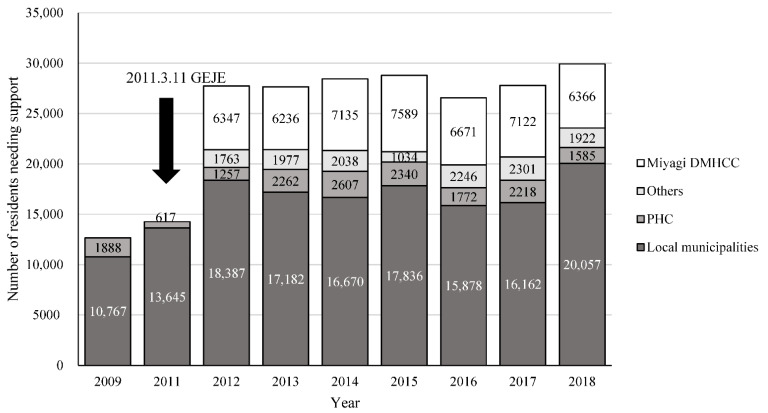
The cumulative number of mental health support cases in Miyagi prefecture from 2009 to 2017 across different organizations. GEJE, Great East Japan Earthquake; PHC, Public Health Center.

On 11 March 2011, an earthquake with a magnitude of 9.0 and a tsunami hit the eastern part of Japan; 19,630 people died, and 6230 were injured. A further 2569 people were reported missing [17] and approximately 400,000 people were evacuated to emergency shelters right after the disaster. Before the disaster, the highly rural areas affected by the earthquake did not have adequate mental health services. As the Japanese government was forced to cope with the situation at hand in a relatively short period, the DMHCCs were established as community health recovery centers in the three prefectures of the Tohoku region. The Miyagi DMHCC was established as a long-term mental health system in December 2011, eight months after the disaster. The center comprised 40 staff members across three branches. The team consisted of various mental health professionals: psychiatrists, psychiatric social workers, public health nurses, psychologists, nurses, and occupational therapists. The Miyagi DMHCC supported residents through home visits, telephone consultations, and counseling for walk-in visitors. The Japanese government invested approximately 2,500,000 US dollars (300,000,000 Yen) a year in the Miyagi DMHCC and the initial span of the entire project was 10 years. Figure 1 presents the annual number of residents who needed mental health support from 2009 to 2018, based on the data from the Miyagi prefecture. The number of residents who needed mental health support in the Miyagi prefecture drastically increased from 14,262 in 2011 to 27,754 in 2012. This shows that the number of residents who needed mental health support dramatically increased after the 2011 disaster, and the Miyagi DMHCC continuously followed up with additional residents, from 2012.

Although some existing works reviewed the activities of the DMHCC [28,29], there are no reports on the support needs and the characteristics of residents who needed mental health support. Therefore, this study aimed to understand which population needed mental health support through an explanatory analysis of the characteristics of the residents using logistic regression.

## 2. Methods

### 2.1. Study Design

This study was based on the residents’ data who were supported by the Miyagi DMHCC from 2013 to 2018. Since no informed consent was obtained from any individual, personal information was not included in this study. The statement of opting-out was developed on the webpage of the Miyagi DMHCC and procedures as to how to opt-out were also displayed on the webpage. The Miyagi DMHCC developed a brief database in 2013 and recorded its support activities in the form of digital data. The workers of the center confirmed and recorded the residents’ information, including gender, age, area of residence, medical history, and other disaster status. The experiences that could be a psychological burden were confirmed: they had lost loved ones (Yes/No), had been injured (Yes/No), had near-death experiences (Yes/No), lost their community (Yes/No), loss of property other than the house (Yes/No), forced evacuation from home (Yes/No), or lost their jobs (Yes/No). The consultation pathway was considered: whether patients visited by themselves, or were referred by family members, by neighbors, by workplace, through a health survey by local municipalities and public health care centers, by temporary housing supporters, by medical resources, or by welfare facilities, among others. 

This study was approved by the Miyagi DMHCC Ethics Committee (Ethical Approval Reference No. 2020-03).

### 2.2. Participants

**Figure 2:**
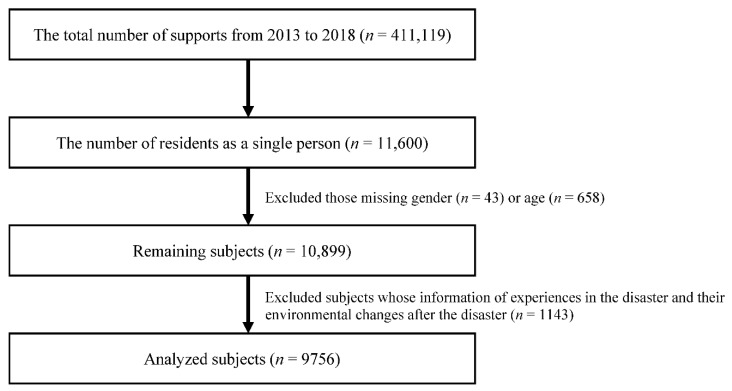
Sample selection from the Miyagi DMHCC database.

The total number of supports from 2013 to 2018 was 411,119, counted as a multiple because many were supported several times. The basic characteristics, life backgrounds before the disaster, and experiences that could be a psychological burden during the disaster of the residents have shown in Table 1. The number of residents identified as individuals was 11,600. The data of individuals for whom gender information (*n* = 43) and age (*n* = 658) were unavailable were excluded from the analysis. Furthermore, the residents whose information of experiences in the disaster and their environmental changes after the disaster was incomplete were also excluded (*n* = 1143). Thus, 9756 residents were eligible for analysis (Figure 2).

### 2.3. Statistical Analyses

We performed chi-squared tests and two multivariate logistic regression models to examine the characteristics of residents in two significant pathways and to identify the odds ratios (OR) and the corresponding 95% confidence intervals (CI). We focused on two major pathways (seeking help by themselves and home visits according to the request from local municipalities) to understand the trend of the residents. Two multivariate logistic regressions were performed: the dependent factor was visiting the center to seek help by themselves (Yes/No) in the first analysis (Table 2) and was being supported by home visits through the health survey (Yes/No) in the second one (Table 3). In both, the independent factors were sex, year, age group, life background before the disaster, and experiences that could be a psychological burden: lost loved ones, had been injured, had a near-death experience, lost their community, and lost their job. A significance value of *p* < 0.05 was considered statistically significant. Data were analyzed using SPSS (version 23.0; IBM SPSS, Armonk, NY, USA).

## 3. Results

The total number of residents who needed support was 9756 in six years, from 2013 to 2018 (1882 in 2013; 2372 in 2014; 2206 in 2015; 1269 in 2016; 1067 in 2017; and 960 in 2018). There was no significant difference among residents based on gender: the number of males and females were 4326 (44.3%) and 5430 (55.7%), respectively. There was no significant difference in the mean age of males (55.6; SD ± 18.5) and females (57.1; SD ± 18.6), those age range was from one to 97. The chi-squared test of independence revealed significant differences according to gender in terms of the annual number of individuals supported by the center. Furthermore, the chi-squared test of independence revealed significant differences between individuals who experienced a psychological burden and those who did not. The test also demonstrated significant differences among each of the experiences.

**Figure 3:**
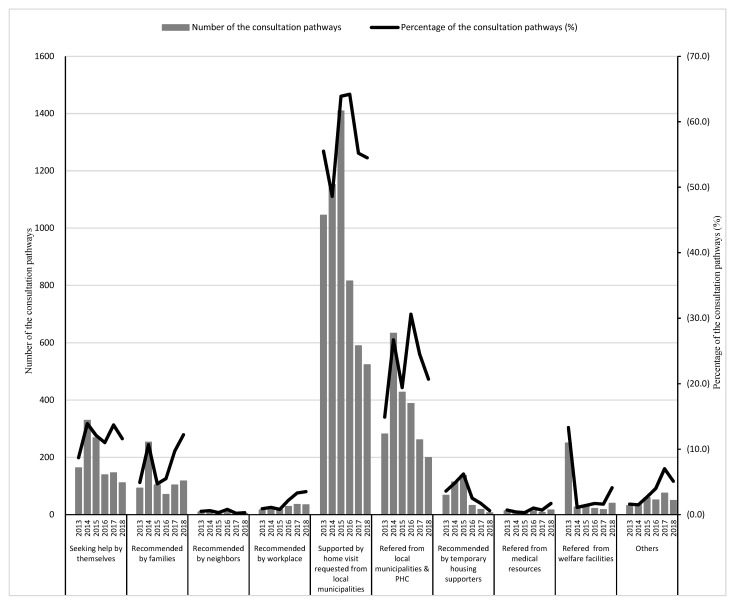
Number and percentage of the consultation pathways used. PHC, Public Health Center.

Residents were connected to the Miyagi DMHCC through various pathways. Figure 3 shows the number and percentage of individuals who chose a particular pathway to access the health services offered by the center. There were two major pathways: seeking help by themselves and being supported by home visits requested from local municipalities through the survey. Many of the local municipalities, which were severely affected, conducted various types of health surveys. Additionally, workers visited all residents if the population was small. Although they understood where the residents who needed some help were located and recommended visiting some resources, many of them refused for several reasons. Therefore, the local municipalities requested the center to visit these residents regularly.

Table 2 shows the result of the multivariate logistic regression analysis for residents who visited the center to seek help by themselves. It was observed that males tended to visit the center by themselves (OR 1.55, 95%CI 1.38–1.80). Residents aged 20–30 years old (OR 3.39, 95%CI 2.27–5.07) and 30–40 years old (OR 2.48, 95%CI 1.69–3.65) tended to visit the center. If the residents lived alone, they did not seek help by themselves (OR 0.75, 95%CI 0.65–0.87). Furthermore, those who had a history of visiting psychiatric facilities (OR 2.04, 95%CI 1.77–2.34) and lost their job due to the disaster (OR 1.53, 95%CI 1.21–1.93) was significantly associated with aid-seeking behavior.

Table 3 shows the result of multivariate logistic regression analysis for residents who were supported by home visits through the survey. It was observed that males did not tend to prefer home visits (OR 0.71, 95%CI 0.64–0.77). Regarding age group, almost all age groups were less associated with this support: less than 20 years old (OR 0.03, 95%CI 0.02–0.04), 20–30 years old (OR 0.25, 95%CI 0.21–0.31), 30–40 years old (OR 0.39, 95%CI 0.33–0.46), 40–50 years old (OR 0.52, 95%CI 0.45–0.60), and 50–60 years old (OR 0.67, 95%CI 0.58–0.77). Residents who lived alone (OR 1.41, 95%CI 1.27–1.56) and lost their community (OR 1.54, 95%CI 1.38–1.73) majorly received home visits. The residents who had a history of visiting psychiatric facilities (OR 0.19, 95%CI 0.17–0.21) and had been injured in the disaster (OR 0.33, 95%CI 0.25–0.44) did not associate with receiving home visits.

## 4. Discussion

After the GEJE disaster, an increase in the demand for mental health services was expected, as a vast number of residents experienced psychological trauma due to the disaster. Owing to the lack of mental health resources in the disaster-affected area in the aftermath of the 2011 earthquake, local municipalities and public health centers faced difficulties in providing residents with adequate treatment. The number of residents who needed mental health support dramatically increased after the 2011 disaster, and the Miyagi DMHCC continuously followed up on additional residents (Figure 1). A report on post-disaster mental health services revealed that, on a few occasions, residents sought psychological support by themselves right after a disaster, while intervention from mental health systems was needed in subsequent phases [11,30]. As the tendency observed in the Miyagi prefecture was similar to the aforementioned report, immediate preparation was required to prevent a future inability to provide the necessary care to meet the increasing demand for consultation. In response to this growing need for mental health services, the Miyagi prefecture local administration collaborated with local municipalities to conduct a health survey of the affected residents who were financially supported by them [31,32]. The Miyagi DMHCC collaborated with the local municipalities to support the residents who could not visit mental health resources through home visits and provided them with appropriate further support.

The residents who visited the center and sought help by themselves were not a large percentage compared to the overall support activities of the center. In our study, males, a relatively younger age group (20–40 years old), who had lost their jobs had a significant association with help-seeking behavior. There is a possibility that some men, especially those of working age, struggled to reconstruct their life because they lost their job, and therefore started seeking mental health support by themselves. The number of completely unemployed individuals was estimated to be approximately 150,000 to 200,000 in several years following the disaster, and the largest population was the primary industry such as fishing and agriculture; approximately 40% of them became unemployed [33]. This result might be due to the structure of society; the labor situation in Japan is such that the employment rate of females is low, and many men are solely responsible for their household finances. To date, no study has addressed help-seeking behaviors after disasters according to the background of the residents. In a male-dominant society such as Japan, since there is a high need for mental health support for working-age men for several years following the disaster, the system can identify unemployed men with psychological risk in the workplace or Public Employment Security Office. After massive disasters, the impact on unemployment and economic status must be paid attention to, especially for working-age men, according to the characteristics of the affected communities.

In our study, the younger residents did not receive home visits, and the residents who lived alone and lost their community were highly associated with receiving home visits. There was a possibility that isolated older females were likely to be found at higher psychological risk by the health survey and were provided with support in the first several years following the disaster. Evidence from a previous health survey indicates that many older individuals lead an isolated life in temporary housing, which is a major issue that needs to be addressed [31]. These results indicate that public health officials and mental health professionals must pay attention to the isolation experienced by older adults following a large disaster. Several suggestions to prevent the isolation of such individuals can be made following the results of this study. First, temporary housing (such as the temporary post-disaster container housing provided by local governments) should be built while considering the accommodation of mixed age groups to promote mutual aid between the residents. If many of the residents in temporary housing are older adults, creating a supportive environment is more challenging. The structure through which residents of all ages support each other is a vital concept for temporary communities. Second, local governments should consistently aim to understand the needs of older people who may be isolated in their communities, and long-term mental health care centers should keep in touch with such people after disasters. 

The residents who experienced visiting psychiatric facilities were significantly associated with seeking help by themselves and did not tend to receive home visits in this study. Since some major disasters make it difficult to access mental health resources and routine treatment is interrupted [34,35], the Japanese government allowed the mental health support team to distribute medication without prescription. These teams connected these residents to the mental health facilities in the disaster-affected areas. Natural disasters may cause an additional psychological burden for the residents with schizophrenia [36], PTSD [37], depression [38], and substance use disorders [39], and some of their mental health conditions relapse following the disaster. North [2] pointed out that the delivery of mental health services to residents with pre-existing mental illness plays a key role in the post-disaster affected areas. The residents with pre-existing mental disorders have to be paid attention to after massive disasters. Some research mentioned that mental health needs may not be high right after disasters, but can exist for months after disasters [2,12].

## 5. Conclusions

The number of residents who needed mental health support increased after the 2011 earthquake, and the Miyagi DMHCC was established in response to the disaster to provide long-term community support. Working-age men sought help by themselves due to unemployment or economic status, and isolated older females who were evaluated to be at high risk through the survey were supported by home visits. In addition, residents with pre-existing mental disorders were supported by the emergency support team and connected to the Miyagi DMHCC smoothly.

As post-disaster reconstruction efforts change phases, community residents face diverse issues and need specialized mental health support. It is necessary to observe these changes and provide appropriate support at the correct time. Further research regarding the increase in specific mental illnesses and budget allocation for mental health is needed. 

### Limitations

Despite its clear contributions, this article has several limitations. First, since the subjects we analyzed included only the residents who were connected to the center, they were not representative of the communities. Therefore, the study was only an explanatory analysis of the residents who were supported by the center and is limited generalizability. Second, since any standardized psychological rating scales were not used in this study, the data might not correctly reflect the psychological status of the residents. Third, because the Miyagi DMHCC is not a medical organization, it mainly connected the residents with pre-existing community resources. As a result, the researchers were unable to ensure that all the individuals seeking support recovered and could not analyze the extent to which their efforts contributed to the individuals’ recovery. Finally, the residents on whom long-term mental health care centers should focus may differ, depending on the structure of the communities before disasters. The problems faced by communities may be different from those explored in this study if the main industry of the region is urbanized, the residents are younger, and the employment rate of the females is higher. The characteristics of the Japanese society, the aging population, and the low female employment rate might have affected this study’s results.

## Figures and Tables

**Table 1:** The basic characteristics, life backgrounds before the disaster, and experiences that could be a psychological burden during the disaster of the residents.

	2013		2014		2015		2016		2017		2018			
	(*n* = 1882)		(*n* = 2372)		(*n* = 2206)		(*n* = 1269)		(*n* = 1067)		(*n* = 960)			
	No	%	No	%	No	%	No	%	No	%	No	%	*p*-Value	χ2
No														
Male	729	(38.7)	1084	(45.7)	994	(45.1)	563	(44.4)	476	(44.6)	480	(50.0)	<0.001	38.7
Female	1153	(61.3)	1288	(54.3)	1212	(54.9)	706	(55.6)	591	(55.4)	480	(50.0)		
Age group														
Less than 20 years old	37	(2.0)	70	(3.0)	57	(2.6)	55	(4.3)	79	(7.4)	106	(11.0)	<0.001	4193.1
20–30 years old	110	(5.8)	102	(4.3)	117	(5.3)	71	(5.6)	73	(6.8)	61	(6.4)		
30–40 years old	189	(10.0)	193	(8.1)	199	(9.0)	150	(11.8)	135	(12.7)	86	(9.0)		
40–50 years old	283	(15.0)	304	(12.8)	295	(13.4)	163	(12.8)	159	(14.9)	127	(13.2)		
50–60 years old	313	(16.6)	334	(14.1)	322	(14.6)	175	(13.8)	145	(13.6)	146	(15.2)		
60–70 years old	434	(23.1)	636	(26.8)	529	(24.0)	292	(23.0)	230	(21.6)	205	(21.4)		
70 years old or more	516	(27.4)	733	(30.9)	687	(31.1)	363	(28.6)	246	(23.1)	229	(23.9)		
Life backgrounds before the disaster														
Lived alone Yes (vs. No)	1495	(79.4)	1742	(73.4)	1623	(73.6)	926	(73.0)	790	(74.0)	705	(73.4)	<0.001	28.8
Had a history of visiting psychiatric facilities Yes (vs. No)	335	(17.8)	814	(34.3)	380	(17.2)	211	(16.6)	198	(18.6)	176	(18.3)	<0.001	297.2
Experiences that could be a psychological burden during the disaster														
Lost loved ones Yes (vs. No)	276	(14.7)	393	(16.6)	250	(11.3)	157	(12.4)	102	(9.6)	107	(11.1)	<0.001	50.5
Had been injured Yes (vs. No)	46	(2.4)	145	(6.1)	87	(3.9)	24	(1.9)	11	(1.0)	12	(1.3)	<0.001	102.8
Had a near-death experience Yes (vs. No)	394	(20.9)	545	(23.0)	335	(15.2)	191	(15.1)	102	(9.6)	105	(10.9)	<0.001	155.0
Lost of their community Yes (vs. No)	421	(22.4)	726	(30.6)	335	(30.6)	322	(25.4)	234	(21.9)	241	(25.1)	<0.001	68.1
Lost their job Yes (vs. No)	171	(9.1)	287	(12.1)	335	(5.7)	65	(5.1)	44	(4.1)	44	(4.6)	<0.001	128.1

No = the number of residents in the year; % = the proportion of the residents in the year.

**Table 2:** Factors related to the residents who visited the center by themselves.

	Seeking Help by Themselves (*n* = 1156)		Others (*n* = 8600)				
	No	%	No	%	*p*-Value	OR	95%CI
Male	411	(35.6)	745	(64.4)	<0.01	1.55	(1.38–1.80)
Year					0.05	1.04	(1.00–1.09)
20–30 years old	142	(12.3)	1014	(87.7)	<0.01	3.39	(2.27–5.07)
30–40 years old	203	(17.6)	953	(82.4)	<0.01	2.48	(1.69–3.65)
40–50 years old	221	(19.1)	935	(80.9)	0.01	1.63	(1.11–2.40)
50–60 years old	158	(13.7)	998	(86.3)	0.55	1.13	(0.76–1.67)
60–70 years old	219	(18.9)	937	(81.1)	0.79	0.95	(0.65–1.39)
70 years old or more	178	(15.4)	978	(84.6)	0.10	0.72	(0.49–1.06)
Lived alone	834	(72.1)	322	(27.9)	<0.01	0.75	(0.65–0.87)
Had a history of visiting psychiatric facilities	427	(36.9)	729	(63.1)	<0.01	2.04	(1.77–2.34)
Lost loved ones	111	(9.6)	1045	(90.4)	0.05	0.80	(0.64–1.00)
Had been injured	18	(1.6)	1138	(98.4)	0.01	0.51	(0.31–0.86)
Had a near-death experience	187	(16.2)	969	(83.8)	0.55	0.94	(0.78–1.14)
Lost their community	250	(21.6)	906	(78.4)	0.53	0.95	(0.81–1.12)
Lost their job	117	(10.1)	1039	(89.9)	<0.01	1.53	(1.21–1.93)

No = the number of the residents in the year; % = the proportion of the residents; OR = odds ratio; 95%CI = 95% confidential intervals.

**Table 3:** Factors related to the residents who were supported by home visits through the screening.

	Supported by Home Visits (*n* = 5533)		Others (*n* = 4223)				
	No	%	No	%	*p*-Value	OR	95%CI
Male	2918	(52.7)	2615	(47.3)	<0.01	0.71	(0.64–0.77)
Year					<0.01	1.07	(1.04–1.10)
Less than 20 years old	36	(0.7)	5497	(99.3)	<0.01	0.03	(0.02–0.04)
20–30 years old	191	(3.5)	5342	(96.5)	<0.01	0.25	(0.21–0.31)
30–40 years old	434	(7.8)	5099	(92.2)	<0.01	0.39	(0.33–0.46)
40–50 years old	633	(11.4)	4900	(88.6)	<0.01	0.52	(0.45–0.60)
50–60 years old	808	(14.6)	4725	(85.4)	<0.01	0.67	(0.58–0.77)
60–70 years old	1475	(26.7)	4058	(73.3)	0.01	0.83	(0.73–0.95)
Lived alone	4214	(76.2)	1319	(23.8)	<0.01	1.41	(1.27–1.56)
Had a history of visiting psychiatric facilities	515	(9.3)	5018	(90.7)	<0.01	0.19	(0.17–0.21)
Lost loved ones	703	(12.7)	4830	(87.3)	0.07	0.88	(0.76–1.01)
Had been injured	107	(1.9)	5426	(98.1)	<0.01	0.33	(0.25–0.44)
Had a near-death experience	882	(15.9)	4651	(84.1)	0.50	0.95	(0.83–1.09)
Lost their community	1719	(31.1)	3814	(68.9)	<0.01	1.54	(1.38–1.73)
Lost their job	393	(7.1)	5140	(92.9)	0.58	0.95	(0.79–1.14)

No = the number of the residents in the year; % = the proportion of the residents; OR = odds ratio; 95%CI = 95% confidential intervals.

## Data Availability

The data presented in this study are available on request from the corresponding author. The data are not publicly available due to privacy issues.
